# A Technological Tool Aimed at Self-Care in Patients With Multimorbidity: Cross-Sectional Usability Study

**DOI:** 10.2196/46811

**Published:** 2024-04-05

**Authors:** Rodrigo Medina-García, Juan A López-Rodríguez, Cristina María Lozano-Hernández, Verónica Ruiz Bejerano, Paride Criscio, Isabel Del Cura-González, TeNDER Atención Primaria Group

**Affiliations:** 1 Primary Care Research Unit Madrid Health Service Madrid Spain; 2 General Ricardos Primary Health Care Centre Madrid Health Service Madrid Spain; 3 Interuniversity Doctoral Program in Epidemiology and Public Health Rey Juan Carlos University Alcorcón, Madrid Spain; 4 Biosanitary Research and Innovation Foundation of Primary Care Madrid Spain; 5 Research Network on Chronicity, Primary Care and Health Promotion Madrid Spain; 6 Department of Medical Specialties and Public Health Faculty of Health Sciences Rey Juan Carlos University Alcorcón, Madrid Spain; 7 Visual Telecommunications Application Research Group Universidad Politécnica de Madrid Madrid Spain; 8 DataWizard Srl Rome Italy

**Keywords:** user-centered design, multimorbidity, comorbid, self-care, medical informatics, primary health care, chronic disease, chronic condition, chronic illness, primary care, usability, telemedicne, telehealth, information and communication technologies, ICT, digital health, eHealth, human-computer interaction

## Abstract

**Background:**

Information and communication technologies (ICTs) have been positioned as useful tools to facilitate self-care. The interaction between a patient and technology, known as usability, is particularly important for achieving positive health outcomes. Specific characteristics of patients with chronic diseases, including multimorbidity, can affect their interaction with different technologies. Thus, studying the usability of ICTs in the field of multimorbidity has become a key element to ensure their relevant role in promoting self-care.

**Objective:**

The aim of this study was to analyze the usability of a technological tool dedicated to health and self-care in patients with multimorbidity in primary care.

**Methods:**

A descriptive observational cross-sectional usability study was performed framed in the clinical trial in the primary care health centers of Madrid Health Service of the TeNDER (Affective Based Integrated Care for Better Quality of Life) project. The TeNDER technological tool integrates sensors for monitoring physical and sleep activity along with a mobile app for consulting the data collected and working with self-management tools. This project included patients over 60 years of age who had one or more chronic diseases, at least one of which was mild-moderate cognitive impairment, Parkinson disease, or cardiovascular disease. From the 250 patients included in the project, 38 agreed to participate in the usability study. The usability variables investigated were effectiveness, which was determined by the degree of completion and the total number of errors per task; efficiency, evaluated as the average time to perform each task; and satisfaction, quantified by the System Usability Scale. Five tasks were evaluated based on real case scenarios. Usability variables were analyzed according to the sociodemographic and clinical characteristics of patients. A logistic regression model was constructed to estimate the factors associated with the type of support provided for task completion.

**Results:**

The median age of the 38 participants was 75 (IQR 72.0-79.0) years. There was a slight majority of women (20/38, 52.6%) and the participants had a median of 8 (IQR 7.0-11.0) chronic diseases. Thirty patients completed the usability study, with a usability effectiveness result of 89.3% (134/150 tasks completed). Among the 30 patients, 66.7% (n=20) completed all tasks and 56.7% (17/30) required personalized help on at least one task. In the multivariate analysis, educational level emerged as a facilitating factor for independent task completion (odds ratio 1.79, 95% CI 0.47-6.83). The median time to complete the total tasks was 296 seconds (IQR 210.0-397.0) and the median satisfaction score was 55 (IQR 45.0-62.5) out of 100.

**Conclusions:**

Although usability effectiveness was high, the poor efficiency and usability satisfaction scores suggest that there are other factors that may interfere with the results. Multimorbidity was not confirmed to be a key factor affecting the usability of the technological tool.

**Trial Registration:**

Clinicaltrials.gov NCT05681065; https://clinicaltrials.gov/study/NCT05681065

## Introduction

Multimorbidity, which is generally defined as the presence of two or more simultaneous chronic diseases in a patient, is a major challenge for health systems [1]. In the European Union, up to 50 million people are estimated to have multimorbidity [2]. Barnett et al [3] estimated a multimorbidity prevalence of 64.9% among patients aged 65-84 years and of 81.5% for those 85 years or older [3]. In recent years, patient-centered care models [4] and, more specifically, interventions aimed at self-care education have made it possible to optimize how patients with multimorbidity manage their chronic diseases [5]. This type of intervention makes it easier for patients to identify their health-problem needs and to identify techniques that can help them make decisions, take appropriate actions, and modify them as they present changes in their diseases [6].

Information and communication technologies (ICTs) have been positioned as useful tools to facilitate self-care [7]. The different self-care strategies using ICTs include those dedicated to the monitoring of biometric parameters through wearable technologies or portable devices and mobile apps [8,9]. To achieve positive health outcomes from these interventions, the interaction between a patient and technology is particularly important. The description of this interaction between the technology, the specific tasks to be developed, and the end user is a property known as usability [10].

Research on usability has grown in parallel with the development of ICTs in health [11]. Reports from international organizations such as the 2012-2020 eHealth Action Plan of the European Commission [12] and the World Health Organization Global Strategy on Digital Health 2020-2025 [13] summarized the importance of the development of technological tools that take into account their interaction with the special conditions of older adults. The study of usability can help determine the reasons for low patient adherence and adoption of a specific technological tool. The improvements in usability could facilitate interaction through several mechanisms: reducing anxiety related to the use of new tools, increasing accessibility and distribution among a greater number of users, and reducing the possible risks derived from misuse [10].

In evaluating usability, the International Organization for Standardization (ISO) 9126 standard [14] assesses the quality of the product [15] and the ISO 9241 standard focuses on processes, referred to as “the extent to which users in a specific environment can use a product to achieve objectives of effectiveness, efficiency and satisfaction in a particular task” [16]. Thus, usability comprises the effectiveness in usability, defined as the degree of completion [11,17,18] and the total number of errors per task; efficiency in usability, defined as the average time to perform the task; and satisfaction in usability, defined as the degree to which the user’s physical and emotional responses resulting from the use of a product satisfy their needs and expectations. The most commonly used methods for usability evaluation are questionnaires and interviews carried out after the use of the technological tool for a certain period of time.

The systematic reviews of Saeed et al [17] and Zapata et al [18] analyzed the definitions of the ISO standards and the methods used in evaluating the usability of health-related technological tools. Their results indicate that the most frequent usability problems are those related to visual aspects of the system and the ability to learn and use specific features [17,18]. However, because the results are limited to a specific technology and may not be generalizable, their interpretation should take into account the special characteristics of the end users, including their health conditions.

The specific characteristics of patients with chronic diseases can affect their interaction with different technologies. For example, in the usability evaluation studies of an app for diabetes self-management [19,20] and that conducted on an automatic drug dispenser for patients with dementia [21], characteristics of the patients were identified that interact with different aspects of usability. Relatedly, Wildenbos et al [22] differentiated four traits related to aging and chronic diseases that act as barriers to usability: cognitive, physical, motivational, and perceptual. Although the development of technological tools aimed at self-care is increasing, their usability has thus far mainly been evaluated in patients with specific isolated pathologies such as in the previous examples. Research from the perspective of patients with multimorbidity has been increasing in recent years [23,24] but remains insufficient [25], even though multimorbidity is the most common way of reporting chronic diseases in the population over 60 years of age [3].

Thus, studying the usability of ICTs has become a key element in the field of multimorbidity [26] to ensure its relevant role in promoting self-care [7]. Along these lines, the TeNDER (Affective Based Integrated Care for Better Quality of Life) project [27,28] was a multisectoral project funded by Horizon 2020, the EU Framework Programme for research and innovation. The TeNDER project developed an integrated care model to manage multimorbidity in patients with dementia, Parkinson disease, and cardiovascular disease in four European countries: Spain, Germany, Italy, and Slovenia. One of the clinical studies related to the TeNDER project was a multicenter, randomized, parallel-group clinical trial carried out in Spain with the main objective of evaluating the effectiveness of the TeNDER system to improve quality of life in patients with chronic diseases. Secondary aims were to describe the satisfaction of patients and their caregivers and the usability of the TeNDER system [29].

The objective of this study was to analyze the usability of a technological tool (TeNDER) dedicated to health and self-care in patients with multimorbidity in primary care.

## Methods

### Design

This was a descriptive observational cross-sectional study of usability. This study was framed in the clinical trial in the primary care health centers of Madrid Health Service of the TeNDER project (ClinicalTrials.gov NCT05681065) [29].

### Ethical Considerations

This study respects the basic ethical principles of autonomy, beneficence, justice, and nonmaleficence, and its development followed the norms of Good Clinical Practice and the principles enunciated in the latest Declaration of Helsinki (Seoul 2013). The study obtained a favorable report from the Research Ethics Board of the Hospital Universitario 12 de Octubre (20/450) and was approved by the Central Research Commission of the Community of Madrid (PC:39/20). Informed consent was obtained from all participants involved in the study. No camera recording or any other identification was made. Patients were included with an anonymous identifier in the data collection logbook (DCL). All data were processed based on the provisions of the EU General Data Protection Regulation 2016/679 of the European Parliament and the Council (April 27, 2016) and the Organic Law on Data Protection and Guarantee of Digital Rights in the Spanish territory (LOPDGDD 3/2018 of 5 December). Participants did not receive any financial compensation for their participation in the study. The only compensation was that received through the user experience during the use of the technological tool.

### Population and Sample

The study population included patients with one or more chronic diseases recruited from four primary care health centers in the Community of Madrid that had been included in the TeNDER project by their referring professionals [27,28].

Patients over 60 years of age who had visited their health center in the last year and who had any of the following chronic diseases were included: mild-moderate cognitive impairment (Mini-Mental State Evaluation [MMSE] score 19-28 points), Parkinson disease, or cardiovascular disease, which includes patients with heart failure, chronic ischemic heart disease, or atrial fibrillation. Patients with a life expectancy of less than 6 months based on the opinion of their health care professionals, severe mental illness, incapacity for autonomous movement, or an MMSE score of less than 19 points were excluded.

The 250 patients included in the primary care health centers of Madrid Health Service for the TeNDER project were invited to participate via text messages with a mobile instant messaging app. Thirty-eight patients with multimorbidity (≥2 chronic diseases) agreed to participate. Considering an approximate 90% completion rate of tasks in previous usability evaluation studies of monitoring tools [19,25,30], with this sample size, we report a precision of 9.6% with the 95% CI.

### TeNDER Technological Tool

**Figure 1:**
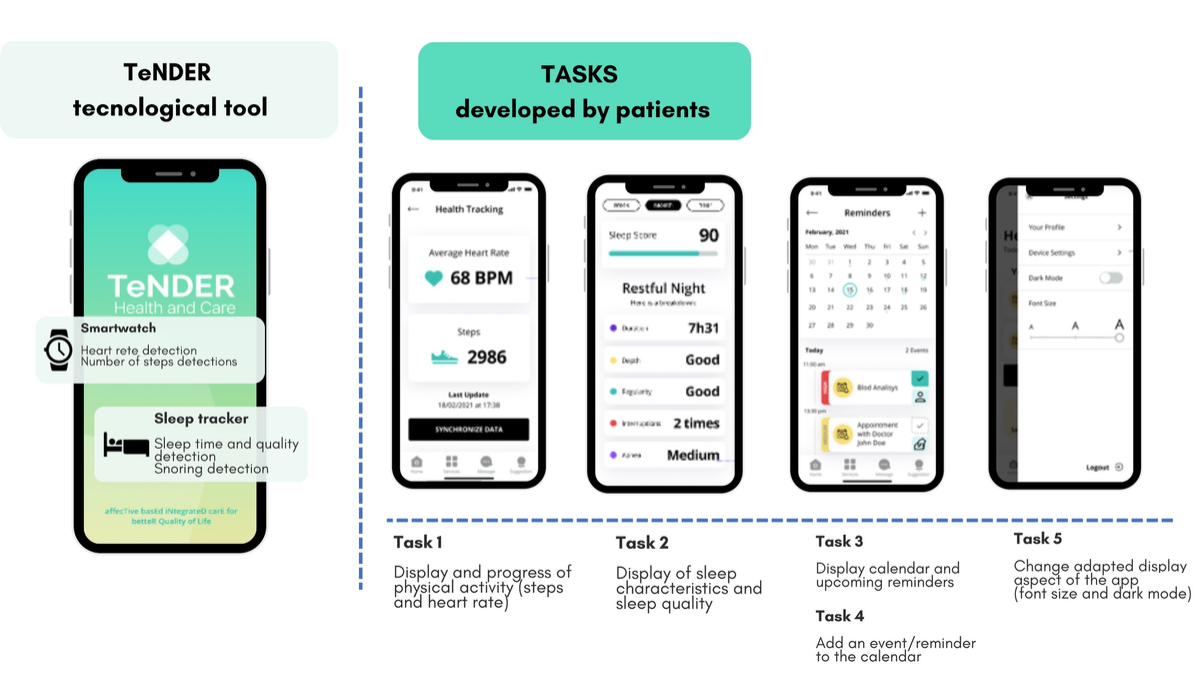
TeNDER technological tool and tasks to be performed by patients for the usability evaluation.

The TeNDER technological tool is a web-based platform that included integrating sensors such as a smartwatch for monitoring physical activity, a sleep tracker to study sleep activity, and a mobile app in which the data collected are displayed and tools for self-management are offered (Figure 1). All of the TeNDER ecosystem technology was developed through a co-design process with all relevant stakeholders using a patient-centered approach. During the project, the functionalities and the mobile app were validated and released after user validation within an incremental development approach, ensuring a feedback framework that provided iterative refinement and improvements of the mobile app.

### Variables

The main variable of the study was usability effectiveness, which was determined by the degree of completion and the total number of errors per task. Five tasks were designed using the TeNDER system (Textbox 1). The tasks to be evaluated were based on real case scenarios to simulate how patients would interact with the system in a real-life situation according to the care and self-management process based on the main functionalities of the TeNDER app [29]. The tasks were validated by a panel of three health professionals with experience in the study of usability to verify the accuracy of the content and the context. The degree of completion of the tasks was coded using three categories: (0) not completed when the subject was unable to complete the task (inability to progress or to request advanced help or interruption in task execution), (1) completed with personalized help in the form of comments or directed indications, and (2) completed independently when the user was able to carry out the task either without any help from the person in charge of the test or with the aid of minor indications. An error was coded when the subject made errors that could not be solved or that prevented further progress.

**Tasks evaluated for the usability study.**

Task 1: Display and progress of physical activity (steps and heart rate).

Task 2: Display of sleep characteristics and sleep quality.

Task 3: Display calendar and upcoming reminders.

Task 4: Add an event/reminder to the calendar.

Task 5: Change-adapted display aspects of the app (font size and dark mode).

As secondary variables, usability efficiency was determined by timing each individual task and calculating the average time in each task. Usability satisfaction was quantified by administering the System Usability Scale (SUS) (Multimedia Appendix 1) in its Spanish-validated version [31]. For this scale, the global score ranges from 0 to 100, where higher values indicate greater usability satisfaction. According to Bangor et al [32], SUS scores of 70-100 indicate acceptable, whereas scores of 0-50 indicate not acceptable; scores between 50 and 70 are considered to indicate marginally acceptable results.

Sociodemographic variables collected included age, sex, and education level, and clinical variables included type and number of chronic diseases. Chronic pathologies were identified according to the proposals in the O’Halloran classification [33] (Multimedia Appendix 2).

Technology-related variables included previous use of touch screens and the affinity for technology interaction (ATI) scale [34]. For this scale, the global score ranges between 1 and 6, where higher values indicate a greater affinity for the technology (Multimedia Appendix 3).

### Data Collection

The variables were collected by interview with the patient in consultation with their referring professional and were recorded in an electronic DCL designed ad hoc with the Research Electronic Data Capture (REDCap) tool hosted on the secure storage server of the institution. REDCap is a secure, web-based software platform designed to support data capture for research studies [35,36].

The patients received the TeNDER technological tool. The usability study was carried out 48 hours afterward based on the execution of tasks in a face-to-face session with a member of the research team who could provide assistance. To record the variables that measure usability, a real-time screen recording of the mobile device was performed during the entirety of task performance. One member of the research team analyzed the recordings. The start and end times were determined from the time the instructions were offered until the moment each task was completed; that information was subsequently transferred to the DCL.

### Statistical Analysis

The categorical variables are described as frequencies and percentages. The quantitative variables are described as medians and IQR, as they were nonnormally distributed for the number of patients under study. The main result variable was the proportion of completed tasks (usability effectiveness) with its 95% CI. As secondary outcome variables, the mean effective time to perform each of the tasks (usability efficiency) and the mean score in the SUS questionnaire (usability satisfaction) were estimated. The association of the different usability components (efficacy, effectiveness, and satisfaction) with the sociodemographic and clinical variables was evaluated using the *χ*^2^ test for categorical variables and the Student *t* test for quantitative variables (the Mann-Whitney *U* test was used for comparison of variables that did not follow a normal distribution). The factors associated with completing the task in an independent manner were analyzed using a multiple logistic regression model with robust estimators. The dependent variable was completing the task autonomously. The independent variables were those found to be statistically significant in the bivariable analyses or variables that are otherwise considered to be clinically important. STATA 14 software was used for all statistical analyses.

## Results

**Figure 2:**
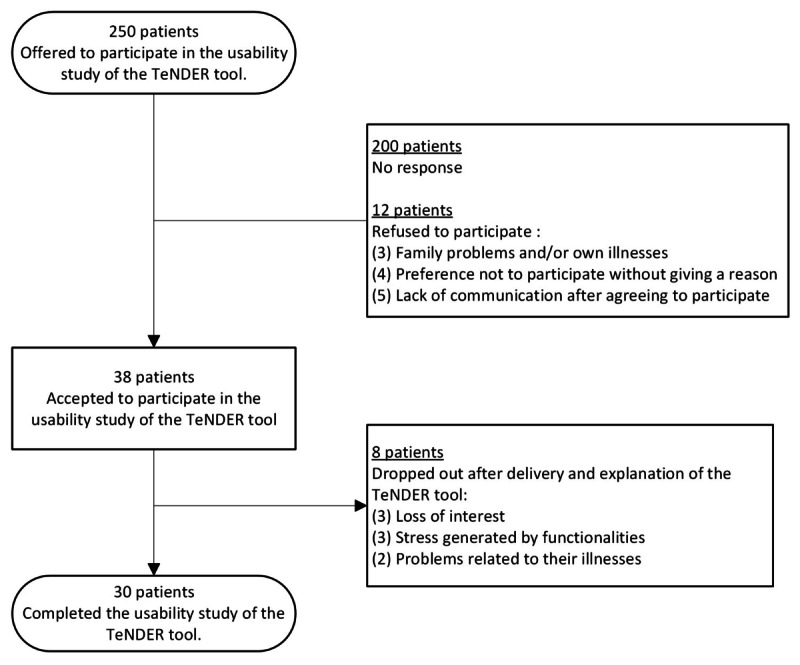
Flowchart of the participants.

Among the 250 patients included in the TeNDER project invited, 38 (15.2%) agreed to participate in this study (Figure 2). There were no differences in sociodemographic characteristics between those who refused to participate and the final sample. Finally, 30 patients completed the usability study.

The median age of the participants was 75 (IQR 72.0-79.0) years and 20/38 (52.6%) were women. With a median of 8 (IQR 7.0-11.0) chronic diseases, 89.5% of the patients had at least one cardiovascular risk factor. Syndromes that include anxiety and depression occurred at significantly different rates between women (11/20, 55%) and men (3/18, 16.7%). A total of 83.8% of the patients had previously interacted with touch screens, and the median result on the ATI scale was 3.4 (IQR 3.0-3.8), which differed between men and women.

The baseline characteristics of the patients are presented in Table 1.

Thirty patients completed the usability study. Among them, 66.7% (20/30) completed all the tasks to be evaluated. All patients were able to complete at least three of the five proposed tasks and 10 patients did not complete at least one task. At least one mistake was made while carrying out the tasks in 28/30 patients. A total of 66.7% (10/15) of the women required personalized help in at least one of the tasks for their completion. The median usability satisfaction in the SUS questionnaire was 55 (IQR 45.0-62.5).

A total of 150 tasks were carried out among all users and 89.3% (134/150) of the tasks were completed. Tasks 1 and 2 were completed 100% (60/60) of the time. Task 4 was completed at a lower proportion than the other tasks (22/30, 73.3%) and presented the highest number of errors (mean 2.5, SD 0.47). Task 3 and task 4 required personalized help to be completed (10/30, 33.3% and 8/30, 26.7%, respectively). The results of the different usability components are shown in Table 2 (also see Multimedia Appendix 4) and the details according to the different tasks are shown in Table 3 (also see Multimedia Appendix 5). The results of the subgroup usability analysis considering patients with cognitive deficits are provided in Multimedia Appendix 6.

In the multivariate analysis of the characteristics of the total tasks evaluated that were completed (Table 4), education level emerged as a facilitating factor to complete the task in an independent manner. Being male, having diseases related to cognition, and age hindered the completion of the task without help, with the latter factor being statistically significant.

**Table 1:** Patient baseline characteristics.

Characteristics				Total		Women		Men		*P* value	
Participants, n (%)				38 (100)		20 (52.6)		18 (47.4)		N/A^a^	
Age (years), median (IQR)				75.0 (72.0-79.0)		75.0 (69.0-80.0)		76.0 (73.0-78.0)		.67	
**Education level, n (%)**											.19
	Up to primary studies		17 (44.7)		8 (40.0)		9 (50.0)				
	Secondary studies		7 (18.4)		6 (30.0)		1 (5.6)				
	Higher education		14 (36.8)		6 (30.0)		8 (44.4)				
Number of chronic diseases, median (IQR)				8.0 (7.0-11.0)		9.0 (8.0-11.5)		8.0 (5.0-10.0)		.05	
**Cardiovascular risk factors, n (%)**											
		Total	34 (89.5)		19 (95.0)		15 (83.3)		.33		
		Arterial hypertension	24 (63.2)		13 (65.0)		11 (61.1)		>.99		
		Lipid metabolism disorders	24 (63.2)		16 (80.0)		8 (44.4)		.04		
		Type 2 diabetes mellitus	13 (34.2)		8 (40.0)		5 (27.8)		.51		
		Overweight/obesity	13 (34.2)		7 (35.0)		6 (33.3)		>.99		
		Cardiovascular disease	28 (73.7)		4 (20.0)		4 (22.2)		>.99		
**Perception problems, n (%)**											
		Total	21 (55.3)		9 (45.0)		12 (66.7)		.21		
		Vision problems	16 (42.1)		8 (40.0)		8 (44.4)		>.99		
Musculoskeletal problems, n (%)				32 (84.2)		19 (95.0)		13 (72.2)		.08	
**Cognition problems, n (%)**											
		Total	21 (55.3)		13 (65.0)		8 (44.4)		.33		
		Cognitive impairment	9 (23.7)		5 (25.0)		4 (22.2)		>.99		
		Anxiety-depression	14 (36.8)		11 (55.0)		3 (16.7)		.02		
Sleep disorders, n (%)				12 (31.6)		4 (20.0)		8 (44.4)		.16	
Previous interaction with touch screens, n (%)				31 (83.8)		15 (78.9)		16 (88.9)		.66	
Affinity for technology interaction scale, median (IQR)				3.4 (3.0-3.8)		3.3 (2.7-3.8)		3.6 (3.2-3.9)		.16	

^a^N/A: not applicable.

**Table 2:** Usability results according to the total number of patients who completed the study and total number of tasks completed.

Usability metric				Total		≤10 CDs^a^		>10 CDs		Women		Men
**Per patient**												
	Number of patients		30		16		14		15		15	
	**Usability effectiveness**											
		Number of tasks completed, median (IQR)	5 (4.0-5.0)		5 (4.0-5.0)		5 (4.0-5.0)		5 (4.0-5.0)		5 (5.0-5.0)	
		At least one task with personalized help, n (%)	17 (56.7)		9 (56.2)		8 (57.1)		10 (66.7)		7 (46.7)	
		All tasks completed independently, n (%)	12 (40.0)		6 (37.5)		6 (42.8)		5 (33.3)		7 (46.7)	
		Number of errors made, median (IQR)	4.0 (2.0-8.0)		5 (2.0-9.0)		4 (2.0-6.0)		6 (3.0-9.0)		3.5 (1.0-5.0)	
	Usability efficiency: time to complete all tasks (seconds), median (IQR)		296.0 (210.0-397.0)		300.0 (236.0-397.0)		284.0 (205.0-431.0)		296.0 (201.0-350.0)		293.5 (211.0-447.0)	
	Usability satisfaction: SUS^b^ questionnaire score, median (IQR)		55.0 (45.0-62.5)		50.0 (45.0-58.8)		61.2 (42.5-70.0)		52.5 (40.0-62.5)		60.0 (47.5-67.5)	
**Tasks**												
	Number of tasks		150		80		70		75		75	
	**Usability effectiveness**											
		Proportion of tasks completed, n (%)	134 (89.3)		71 (88.7)		63 (90.0)		65 (86.7)		69 (92.0)	
		Number of errors per task, mean (SD)	1.0 (1.7)		1.2 (2.0)		0.8 (1.3)		1.4 (2.0)^c^		0.6 (1.2)^c^	
	Usability efficiency: time per task (seconds), mean (SD)		65.4 (92.7)		66.8 (95.1)		63.8 (90.7)		60.1 (79.3)		70.9 (105.1)	

^a^CD: chronic disease.

^b^SUS: System Usability Scale.

^c^*P*=.007. This is the only comparison in which significant differences were found. The table with all *P* values is provided in Multimedia Appendix 4.

**Table 3:** Usability results by task.^a^

Usability metric			Total (N=30)	Women (n=15)	Men (n=15)	≤10 CDs^b^ (n=16)	>10 CDs (n=14)	Up to secondary education (n=18)	Postsecondary education (n=12)
**Task 3**									
	**Usability effectiveness**								
		Number of patients completing the task, n (%)	28 (93.3)	14 (93.3)	14 (93.3)	14 (87.5)	14 (100.0)	16 (88.9)	12 (100.0)
		Completed the task with personalized help, n (%)	10 (33.3)	5 (33.3)	5 (33.3)	5 (31.2)	5 (35.7)	7 (38.9)	3 (25.0)
		Number of errors made, median (IQR)	0.0 (0.0-1.0)	0.0 (0.0-1.0)	0.0 (0.0-1.0)	1.0 (0.0-1.5)	0.0 (0.0-1.0)	0.0 (0.0-1.0)	0.5 (0.0-2.0)
	Usability efficiency: time to perform the task (seconds), median (IQR)		57.0 (33.0-90.0)	40.0 (30.0-70.0)	65.0 (33.0-105.0)	50.0 (23.0-90.0)	58.5 (37.0-90.0)	60.0 (30.0-90.0)	48.5 (34.0-80.0)
**Task 4**									
	**Usability effectiveness**								
		Number of patients completing the task, n (%)	22 (73.3)	9 (60.0)	13 (86.7)	12 (75.0)	10 (71.4)	10 (55.6)^d^	12 (100.0)^d^
		Completed the task with personalized help, n (%)	8 (26.7)	4 (26.7)	4 (26.7)	4 (25.0)	4 (28.6)	5 (27.8)	3 (25.0)
		Number of errors made, median (IQR)	2.0 (0.0-4.0)	3.0 (1.0-5.0)^e^	1.0 (0.0-3.0)^e^	2.0 (0.0-4.0)	1.5 (0.0-3.0)	3.0 (0.0-5.0)	1.0 (0.0-2.0)
	Usability efficiency: time to perform the task (seconds), median (IQR)		182.5 (0.0-280.0)	185.0 (0.0-220.0)	180.0 (137.0-300.0)	186.0 (80.0-265.0)	174.0 (0.0-280.0)	176.0 (0.0-300.0)	186.0 (161.5-225.0)
**Task 5**									
	**Usability effectiveness**								
		Number of patients completing the task, n (%)	26 (86.7)	12 (80.0)	14 (93.3)	15 (93.8)	11 (78.6)	14 (77.8)	12 (100.0)
		Completed the task with personalized help, n (%)	5 (16.7)	2 (13.3)	3 (20.0)	3 (18.8)	2 (14.3)	4 (22.2)	1 (8.3)
		Number of errors made, median (IQR)	1.0 (0.0-2.0)	1.0 (1.0-3.0)^c^	0.0 (0.0-1.0)^c^	0.5 (0.0-2.0)	1.0 (0.0-2.0)	1.5 (0.0-3.0)	0.5 (0.0-1.0)
	Usability efficiency: time to perform the task (seconds), median (IQR)		20.0 (10.0-50.0)	30.0 (6.0- 75.0)	18.0 (10.0-21.0)	20.0 (14.0-75.0)	19.0 (6.0-31.0)	20.5 (10.0-80.0)	19.0 (12.0-27.5)

^a^The usability results for tasks 1 and 2 are not included because they showed 100% effectiveness in usability; *P* values are only indicated for comparisons in which significant differences were found. The table with all *P* values is provided in Multimedia Appendix 5.

^b^CD: chronic disease.

^c^*P*=.01.

^d^*P*=.01.

^e^*P*=.04.

**Table 4:** Factors associated with completing a task in an independent manner.

Associated factors		Odds ratio (95% CI)		*P* value
Male sex		0.81 (0.24-2.74)		.74
Age		0.85 (0.77-0.94)		.002
**Education level**				
	Up to secondary education	Reference	N/A^a^	
	Postsecondary education	1.79 (0.47-6.83)	.39	
Diseases related to cognition		0.18 (0.04-0.81)		.03

^a^N/A: not applicable.

## Discussion

### Main Findings

The usability effectiveness of the TeNDER technological tool was 89.3% (134/150). Overall, 40% (12/30) of the patients completed all tasks independently. Task 4 was completed at a lower proportion than the rest of the tasks (22/30, 73.3%) and presented the highest number of errors (mean 2.5, SD 0.47). The usability efficiency, evaluated as the median time to complete the total tasks, was 296.0 seconds (IQR 210.0-397.0), with an average value per task of 65.4 seconds (SD 92.7). The satisfaction in usability perceived by the patients was acceptable (mean 52.2, SD 16.9). Being male, having diseases related to cognition, and age were factors that hindered the completion of the task without personalized help, among which only age was statistically significant.

### Comparison With Other Studies

The usability effectiveness of the TeNDER technological tool was 89.3%, which is similar to the results of previous studies carried out on different categories of patients for similar technological developments. Sánchez-Morillo et al [30] evaluated the usability of a technological tool aimed at monitoring the symptoms of patients with chronic obstructive pulmonary disease, and Georgsson et al [19] evaluated a system designed for the management of self-care in patients with diabetes. The proportion of tasks completed was 88% and 91%, respectively, despite the opposite characteristics of the participants with respect to the level of education and affinity for technology in each of the studies. As in our study, the degree of task complexity could have been adapted to the characteristics of the potential users: tasks 1 and 2 were completed by all patients, whereas the rest of the tasks, of greater complexity, were completed by only those with higher education. For those who did not have higher education, the task completion rate reached up to 55.6%. These differences in the use of technology depending on the level of education have been confirmed in previous studies [37].

Despite the high proportion of completed tasks, 56.7% of the patients required personalized help to complete at least one of the tasks. Older age and cognition-related diseases were risk factors for requiring personalized help to complete the tasks. Previous experience in evaluating the usability of a computerized system for self-care management aimed at patients with chronic diseases yielded similar percentages of effectiveness in usability and help for task completion [25], which points to the importance of having family members or professionals assist patients with chronic diseases to interact with a mobile app [38].

The median value for usability satisfaction was 55.0 (IQR 45.0-62.5), which is a low marginal score over not acceptable [32]. Ligons et al [21] obtained similar results and indicated that the degree of response in satisfaction with a technology or system may not be related to the ability for the completion of its tasks. That is, patients may be able to complete tasks without knowing why they have completed them or how they can benefit from them in their day-to-day lives. Other studies, including that of Sánchez-Morillo et al [30], suggested that high levels of satisfaction may be caused by the presence of qualified professionals who assisted during the usability evaluation.

The median age of the patients in our study was 75 years and a high degree of multimorbidity was notable, with a median of up to eight chronic diseases. Previous studies have analyzed the usability of a technology from the perspective of patients with a chronic index disease in particular [19,20,30,39]. For example, Wildenbos et al [22] analyzed how chronic diseases can affect the usability of technological tools. Thus, a single chronic disease can be the cause of physical, cognitive, and perception barriers [22]. Medical conditions that could favor the appearance of these barriers are represented in our study: diabetes, cardiovascular disease, cognitive impairment, and vision problems. However, as in previous studies, no differences were found in the different aspects of usability based on the number of chronic diseases.

Only 38 patients out of a total of 250 who signed prior informed consent agreed to participate in this usability study. It should be noted that a mobile instant messaging app was used as the method of offering participation and there was a nonresponse rate of 80% (200/250). This means of communication, although common in current society, could have caused a lack of confidence or security in patients [40]. Among the 38 patients who agreed to participate, 6 (15.2%) decided to leave the study as a result of the stress generated by the proposed tasks or due to lack of interest. Few previous usability studies have reported the number of losses [41], perhaps due to the small number of patients involved. Wildenbos et al [22] mentioned lack of motivation as a key element to achieving acceptance of technology by older people. The benefits of using a technology should be made evident quickly and easily; otherwise, feelings of frustration and of giving up its use are likely. In a time-limited usability evaluation, these benefits are not evident, and their nonparticipation can help to avoid feelings of uncertainty, wasting the time invested in learning a technology, or the shame of making mistakes.

Differences based on sex in the use of ICTs have been described in previous studies [37,42]. Among older people, access to technology and their degree of involvement in daily activity is greater in men than in women [37]. These differences are also identified in the different aspects of usability. In our study, the average number of errors committed per task was significantly higher in women (mean 1.4, SD 2.0) than in men (mean 0.6, SD 1.2). In addition, the proportion of women who needed personalized help to complete tasks was higher (10/15, 67%) than that in men (7/15, 47%). These differences have been largely justified by the fact that the labor participation of women has been lower, particularly in computerized jobs due to less training over the years [43].

### Limitations

Although the number of participants in our study is similar to that of other studies and the findings obtained provide valuable information, a larger sample size would provide a larger data set to conduct more sophisticated and detailed statistical analyses. Moreover, given the characteristics of the research, it has not been possible to collect opinions, sensations, and emotions in relation to the technological tool that the patients experienced during task execution. For this reason, including a qualitative methodology such as focus groups [44], think-aloud tasks [45], or a user-centered cognitive walkthrough [20] could provide essential information for understanding the decision-making of patients with multimorbidity when faced with a mobile app aimed at health.

Another limitation identified is the time of tool use being limited to 48 hours. Studies such as those of Tahsin et al [46] and Baek et al [47] showed how usability results can change at different times over longer intervals of use for up to 1 year.

### Conclusions

Although usability effectiveness was high, the poor efficiency and usability satisfaction results suggest that there are other factors that may interfere with these results. Sex and education level can influence the degree of completion of tasks. It has not been possible to show that multimorbidity is a key factor in the usability results of a technological tool.

## Appendix

Multimedia Appendix 1 System Usability Scale (SUS) questionnaire.

Multimedia Appendix 2 Chronic diseases classified according to O’Halloran et al [33].

Multimedia Appendix 3 Affinity for technology interaction (ATI) scale.

Multimedia Appendix 4 Usability results according to the total number of patients who completed the study and total number of tasks completed with complete *P* values for all comparisons (related to Table 2).

Multimedia Appendix 5 Usability results by task with complete *P* values for all comparisons (related to Table 3).

Multimedia Appendix 6 Subgroup analysis considering patients with and without cognitive deficit.

## Abbreviations

ATI: affinity for technology interaction
DCL: data collection logbook
ICT: Information and communication technology
ISO: International Organization for Standardization
MMSE: Mini-Mental State Evaluation
REDCap: Research Electronic Data Capture
SUS: System Usability Scale
TeNDER: Affective Based Integrated Care for Better Quality of Life

## Data Availability

The data sets generated during and/or analyzed during this study are available in the Zenodo repository [48].

## References

[1] Gijsen R, Hoeymans N, Schellevis FG, Ruwaard D, Satariano WA, van den Bos GA (2001). Causes and consequences of comorbidity: a review. J Clin Epidemiol.

[2] (2018). Multimorbidity: a priority for global health research. The Academy of Medical Science.

[3] Barnett K, Mercer SW, Norbury M, Watt G, Wyke S, Guthrie B (2012). Epidemiology of multimorbidity and implications for health care, research, and medical education: a cross-sectional study. Lancet.

[4] Buetow S (2014). Making the improbable probable: communication across models of medical practice. Health Care Anal.

[5] Smith SM, Wallace E, O'Dowd T, Fortin M (2016). Interventions for improving outcomes in patients with multimorbidity in primary care and community settings. Cochrane Database Syst Rev.

[6] Bodenheimer T, Lorig K, Holman H, Grumbach K (2002). Patient self-management of chronic disease in primary care. JAMA.

[7] Leo DG, Buckley BJR, Chowdhury M, Harrison SL, Isanejad M, Lip GYH, Wright DJ, Lane DA, TAILOR investigators (2022). Interactive remote patient monitoring devices for managing chronic health conditions: systematic review and meta-analysis. J Med Internet Res.

[8] Kim BY, Lee J (2017). Smart devices for older adults managing chronic disease: a scoping review. JMIR Mhealth Uhealth.

[9] Linn N, Goetzinger C, Regnaux J, Schmitz S, Dessenne C, Fagherazzi G, Aguayo GA (2021). Digital health interventions among people living with frailty: a scoping review. J Am Med Dir Assoc.

[10] Jordan PW (1998). An Introduction to Usability.

[11] Maramba I, Chatterjee A, Newman C (2019). Methods of usability testing in the development of eHealth applications: a scoping review. Int J Med Inform.

[12] REPORT on the eHealth Action Plan 2012-2020 - Innovative healthcare for the 21st century. European Commission.

[13] Global Strategy on Digital Health 2020-2025. World Health Organization.

[14] Abran A, Khelifi A, Suryn W, Seffah A (2003). Usability meanings and interpretations in ISO standards. Softw Qual J.

[15] (2016). ISO/IEC 25022:2016 Systems and software engineering. Systems and software quality requirements and evaluation (SQuaRE). Measurement of quality in use. International Organization for Standardization (ISO).

[16] (2018). Ergonomics of human-system interaction - Part 11: Usability: Definitions and concepts (ISO 9241-11). Tienda.

[17] Saeed N, Manzoor M, Khosravi P (2020). An exploration of usability issues in telecare monitoring systems and possible solutions: a systematic literature review. Disabil Rehabil Assist Technol.

[18] Zapata BC, Fernández-Alemán JL, Idri A, Toval A (2015). Empirical studies on usability of mHealth apps: a systematic literature review. J Med Syst.

[19] Georgsson M, Staggers N (2016). Quantifying usability: an evaluation of a diabetes mHealth system on effectiveness, efficiency, and satisfaction metrics with associated user characteristics. J Am Med Inform Assoc.

[20] Georgsson M, Staggers N, Årsand E, Kushniruk A (2019). Employing a user-centered cognitive walkthrough to evaluate a mHealth diabetes self-management application: A case study and beginning method validation. J Biomed Inform.

[21] Ligons FM, Mello-Thoms C, Handler SM, Romagnoli KM, Hochheiser H (2014). Assessing the impact of cognitive impairment on the usability of an electronic medication delivery unit in an assisted living population. Int J Med Inform.

[22] Wildenbos GA, Peute L, Jaspers M (2018). Aging barriers influencing mobile health usability for older adults: a literature based framework (MOLD-US). Int J Med Inform.

[23] Doyle J, Murphy E, Kuiper J, Smith S, Hannigan C, Jacobs A, Dinsmore J (2019). Managing multimorbidity: identifying design requirements for a digital self-management tool to support older adults with multiple chronic conditions.

[24] Chaudhry BM, Dasgupta D, Chawla N (2022). Formative evaluation of a tablet application to support goal-oriented care in community-dwelling older adults. Proc ACM Hum Comput Interact.

[25] Or C, Tao D (2012). Usability study of a computer-based self-management system for older adults with chronic diseases. JMIR Res Protoc.

[26] Peute LW, Wildenbos G, Engelsma T, Lesselroth BJ, Lichtner V, Monkman H, Neal D, Van Velsen L, Jaspers MW, Marcilly R (2022). Overcoming challenges to inclusive user-based testing of health information technology with vulnerable older adults: recommendations from a human factors engineering expert inquiry. Yearb Med Inform.

[27] Affective Based Integrated Care for Better quality of life (TeNDER Project). European Commission.

[28] TeNDER.

[29] Lozano Hernández CM, Medina-García R, de Hoyos-Alonso MC, Garrido-Barral A, Minué Lorenzo C, Sanz-Cuesta T, Serrano J, Del Rio Ponce A, Gómez-Gascón T, Del Cura-González I, TeNDER Group Primary Care (2023). Improvement in quality of life with the use of a technological system among patients with chronic disease followed up in primary care (TeNDER Project): protocol for a randomized controlled trial. JMIR Res Protoc.

[30] Sánchez-Morillo D, Crespo M, León A, Crespo Foix LF (2015). A novel multimodal tool for telemonitoring patients with COPD. Inform Health Soc Care.

[31] Sevilla-Gonzalez MDR, Moreno Loaeza L, Lazaro-Carrera LS, Bourguet Ramirez B, Vázquez Rodríguez A, Peralta-Pedrero ML, Almeda-Valdes P (2020). Spanish version of the system usability scale for the assessment of electronic tools: development and validation. JMIR Hum Factors.

[32] Bangor A, Kortum PT, Miller JT (2008). An empirical evaluation of the System Usability Scale. Int J Hum Comput Interact.

[33] O'Halloran J, Miller G, Britt H (2004). Defining chronic conditions for primary care with ICPC-2. Fam Pract.

[34] Franke T, Attig C, Wessel D (2018). A personal resource for technology interaction: development and validation of the Affinity for Technology Interaction (ATI) scale. Int J Hum Comput Interact.

[35] Harris PA, Taylor R, Minor BL, Elliott V, Fernandez M, O'Neal L, McLeod L, Delacqua G, Delacqua F, Kirby J, Duda SN, REDCap Consortium (2019). The REDCap consortium: building an international community of software platform partners. J Biomed Inform.

[36] Harris PA, Taylor R, Thielke R, Payne J, Gonzalez N, Conde JG (2009). Research electronic data capture (REDCap)--a metadata-driven methodology and workflow process for providing translational research informatics support. J Biomed Inform.

[37] Kim J, Lee HY, Christensen MC, Merighi JR (2017). Technology access and use, and their associations with social engagement among older adults: do women and men differ?. J Gerontol B Psychol Sci Soc Sci.

[38] Smith A (2014). Older adults and technology use. Pew Research Center.

[39] Wildenbos GA, Jaspers MWM, Schijven MP, Dusseljee-Peute LW (2019). Mobile health for older adult patients: using an aging barriers framework to classify usability problems. Int J Med Inform.

[40] Kiat BW, Chen W (2015). Mobile instant messaging for the elderly. Proced Comput Sci.

[41] Czaja SJ, Sharit J, Lee CC, Nair SN, Hernández MA, Arana N, Fu SH (2013). Factors influencing use of an e-health website in a community sample of older adults. J Am Med Inform Assoc.

[42] Milagros S, Lidia A, Cecilia C (2020). Mujeres y digitalización. De las brechas a los algoritmos. Instituto de la Mujer y para la Igualdad de Oportunidades. Ministerio de Igualdad.

[43] Hargittai E, Dobransky K (2017). Old dogs, new clicks: digital inequality in skills and uses among older adults. Can J Commun.

[44] Brown W, Yen P, Rojas M, Schnall R (2013). Assessment of the Health IT Usability Evaluation Model (Health-ITUEM) for evaluating mobile health (mHealth) technology. J Biomed Inform.

[45] Bolle S, Romijn G, Smets EMA, Loos EF, Kunneman M, van Weert JCM (2016). Older cancer patients' user experiences with web-based health information tools: a think-aloud study. J Med Internet Res.

[46] Tahsin F, Tracy S, Chau E, Harvey S, Loganathan M, McKinstry B, Mercer SW, Nie J, Ramsay T, Thavorn K, Palen T, Sritharan J, Steele Gray C (2021). Exploring the relationship between the usability of a goal-oriented mobile health application and non-usage attrition in patients with multimorbidity: a blended data analysis approach. Digit Health.

[47] Baek JY, Na SH, Lee H, Jung H, Lee E, Jo M, Park YR, Jang I (2022). Implementation of an integrated home internet of things system for vulnerable older adults using a frailty-centered approach. Sci Rep.

[48] Medina-García R, Lopez-Rodríguez JA A technological tool aimed at self-care in patients with multimorbidity: cross-sectional usability study (complete study data). Zenodo.

