# Supplementary material for: A Technological Tool Aimed at Self-Care in Patients With Multimorbidity: Cross-Sectional Usability Study
Source: JMIR Hum Factors. 2024 Apr 5;11:e46811. doi: 10.2196/46811 (PMC11031692; doi:10.2196/46811)
Supplement: Multimedia Appendix 1 [file humanfactors_v11i1e46811_app1.docx]

# Multimedia Appendix 1 - System Usability Scale (SUS) questionnaire

The questionnaire is designed to be answered after the user’s interaction with the system. It is prepared such that positive and negative statements are alternated to avoid the usual respondent bias.

|  |  |  | Strongly disagree |  |  |  | Strongly agree |
| --- | --- | --- | --- | --- | --- | --- | --- |
| 1. | I think that I would like to use this system frequently |  |  |  |  |  |  |
|  |  |  | 1 | 2 | 3 | 4 | 5 |
| 2. | I found the system unnecessarily complex |  |  |  |  |  |  |
|  |  |  | 1 | 2 | 3 | 4 | 5 |
| 3. | I thought the system was easy to use |  |  |  |  |  |  |
|  |  |  | 1 | 2 | 3 | 4 | 5 |
| 4. | I think I would need the support of a technical person to be able to use this system |  |  |  |  |  |  |
|  |  |  | 1 | 2 | 3 | 4 | 5 |
| 5. | I found the various functions in this system to be well integrated |  |  |  |  |  |  |
|  |  |  | 1 | 2 | 3 | 4 | 5 |
| 6. | I thought there was too much inconsistency in this system |  |  |  |  |  |  |
|  |  |  | 1 | 2 | 3 | 4 | 5 |
| 7. | I would imagine that most people would learn to use this system very quickly |  |  |  |  |  |  |
|  |  |  | 1 | 2 | 3 | 4 | 5 |
| 8. | I found the system very awkward to use |  |  |  |  |  |  |
|  |  |  | 1 | 2 | 3 | 4 | 5 |
| 9. | I felt very confident using the system |  |  |  |  |  |  |
|  |  |  | 1 | 2 | 3 | 4 | 5 |
| 10. | I needed to learn a lot of things before I could get going with this system |  |  |  |  |  |  |
|  |  |  | 1 | 2 | 3 | 4 | 5 |

Analysis:

1. The contribution for odd items (positive statements) is the scale position minus 1, and the contribution for even items (negative statements) is 5 minus the scale position.
2. The overall score is calculated from the sum of all the item scores multiplied by 2.5, and the overall score ranges from 0 to 100.
