# Supplementary material for: A Technological Tool Aimed at Self-Care in Patients With Multimorbidity: Cross-Sectional Usability Study
Source: JMIR Hum Factors. 2024 Apr 5;11:e46811. doi: 10.2196/46811 (PMC11031692; doi:10.2196/46811)
Supplement: Multimedia Appendix 2 [file humanfactors_v11i1e46811_app2.docx]

# Multimedia Appendix 2 - Chronic diseases classified according to O’Halloran.

| **General and nonspecific problems** |
| --- |
| Asthenia/tiredness/weakness (A04); Tuberculosis (A70); Cancer/malignant neoplasm NS (A79); Multiple congenital anomalies NS (A90) |
| **Blood, hematopoietic organs, and immune system (lymphatics, spleen, and bone marrow)** |
| Hodgkin’s disease/lymphomas (B72); Leukemia (B73); Other hematologic malignancies (B74); Nonspecific hematologic benign neoplasms (B75); Hereditary hemolytic anemias (B78); Anemia due to B12 deficiency/folate deficiency (B81); Other/nonspecific anemias (B82); Purpura/coagulation disorders (B83); HIV infection, AIDS (B90) |
| **Digestive system** |
| Viral hepatitis (D72); Malignant neoplasms of the stomach (D74); Malignant neoplasms of the colon/rectum (D75); Malignant neoplasms of the pancreas (D76); Other digestive malignancies NS (D77); Congenital digestive abnormalities (D81); Diseases of the esophagus (D84); Duodenal ulcer (D85); Other peptic ulcers (D86); Diverticular disease of the intestine (D92); Irritable bowel syndrome (D93); Crohn’s disease/ulcerative colitis (D94); Liver diseases NS (D97); Cholecystitis/cholelithiasis (D98); Other digestive diseases (D99) |
| **Eye and ocular adnexa** |
| Eye/adnexal neoplasms (F74); Retinopathy (F83); Degeneration of the macula (F84); Cataract (F92); Glaucoma (F93); Blindness (F94) |
| **Auditory apparatus** |
| Neoplasms of the auditory apparatus (H75); Vertiginous syndromes (H82); Presbycusis (H84); Deafness (H86) |
| **Circulatory system** |
| Rheumatic fever (K71); Cardiovascular neoplasms (K72); Congenital cardiovascular abnormalities (K73); Cardiac ischemia with angina (K74); Acute myocardial infarction (K75); Cardiac ischemia without angina (K76); Heart failure (K77); Atrial fibrillation/flutter (K78); Paroxysmal tachycardia (K79); Cardiac arrhythmia NS (K80); Cardiac/arterial murmurs NS (K81); Heart lung disease (K82); Valvular heart disease (K83); Other heart diseases (K84); Uncomplicated hypertension (K86); Hypertension affecting target organs (K87); Postural hypotension (K88); Transient cerebral ischemia (K89); Ictus/cerebrovascular accident (CVA)/stroke (K90); Cerebrovascular disease (K91); Atherosclerosis/peripheral arterial disease (K92); Pulmonary embolism (K93); Phlebitis and Thrombophlebitis (K94); Varicose veins in the lower extremities (K95) |
| **Locomotor apparatus** |
| Malignant neoplasm of the musculoskeletal system (L71); Congenital abnormalities of the musculoskeletal system (L82); Neck syndromes (L83); Lumbar/thoracic syndrome without pain radiation (L84); Acquired spinal deformity (L85); Lumbar/thoracic syndrome with pain radiation (L86); Rheumatoid arthritis (L88); Hip osteoarthritis (L89); Knee osteoarthritis (L90); Other osteoarthritis (L91); Shoulder syndrome (L92); Epicondylitis (L93); Osteoporosis (L95); Other diseases of the musculoskeletal system (L99) |
| **Nervous system** |
| Other nervous system infections (N73); Malignant neoplasms of the nervous system (N74); Benign neoplasms of the nervous system (N75); Nonspecific neoplasms of the nervous system (N76); Congenital anomalies of the nervous system (N85); Multiple sclerosis (N86); Parkinson’s disease/parkinsonism (N87); Epilepsy (N88); Migraine (N89); Cluster headache (N90); Trigeminal neuralgia (N92); Carpal tunnel syndrome (N93); Neuritis/peripheral neuropathies (N94); Other neurological diseases (N99) |
| **Psychological problems** |
| Chronic alcohol abuse (P15); Dementia (P70); Other organic psychoses (P71); Schizophrenia (P72); Affective psychosis (P73); Anxiety state or disorder (P74); Somatization/conversion disorder (P75); Depressive disorder (P76); Neurasthenia (P78); Phobia/compulsive disorder (P79); Personality disorder (P80); Hyperactivity disorder (P81); Post traumatic stress disorder (P82); Mental retardation (P85); Anorexia nervosa/bulimia (P86); Other psychoses NS (P98); Other psychological disorders (P99) |
| **Respiratory apparatus** |
| Malignant neoplasm of trachea/bronchus/lung/pleura (R84); Other malignant neoplasms respiratory system (R85); Chronic tonsil hypertrophy/infection/adenopathy (R90); COPD (R95); Asthma (R96); Other respiratory system diseases (R99) |
| **Skin and appendages** |
| Malignant neoplasms of the skin (S77); Seborrheic dermatitis (S86); Atopic eczema/dermatitis (S87); Psoriasis (S91); Acne (S96); Other skin diseases (S99) |
| **Endocrine system, metabolism, and nutrition** |
| Malignant thyroid neoplasm (T71); Other endocrine/nonspecific neoplasms (T73); Congenital endocrine/metabolic abnormalities (T80); Goiter (T81); Obesity (T82); Overweight (T83); Hyperthyroidism/thyrotoxicosis (T85); Hypothyroidism/myxedema (T86); Insulin-dependent diabetes (T89); Noninsulin dependent diabetes (T90); Gout (T92); Lipid metabolism disorders (T93); Other endocrine/metabolic/nutritional problems (T99) |
| **Urinary system** |
| Malignant neoplasms of the kidney (U75); Malignant neoplasms of the urinary bladder (U76); Other malignant neoplasms urinary system (U77); Nephrosis/glomerulonephritis (U88); Other urinary problems/diseases (U99) |
| **Family planning, pregnancy, childbirth, and the puerperium** |
| Female infertility/subfertility (W15); Malignant neoplasm connected to pregnancy (W72) |
| **Female genital system and breasts** |
| Pelvic inflammatory disease (X74); Malignant neoplasms of the cervix (X75); Malignant neoplasm of the female breast (X76); Other female genital neoplasms (X77); Other female genital diseases (X99) |
| **Male genital system and breasts** |
| Malignant neoplasms of the prostate (Y77); Other malignant neoplasms, male genital, or breast (Y78); Benign prostatic hypertrophy (Y85) |
