# Supplementary material for: A Technological Tool Aimed at Self-Care in Patients With Multimorbidity: Cross-Sectional Usability Study
Source: JMIR Hum Factors. 2024 Apr 5;11:e46811. doi: 10.2196/46811 (PMC11031692; doi:10.2196/46811)
Supplement: Multimedia Appendix 3 [file humanfactors_v11i1e46811_app3.docx]

# Multimedia Appendix 3 - Affinity for technology interaction (ATI) scale

The nine-item ATI scale is a unidimensional, short, and reliable scale for assessing affinity for technology interaction. It is grounded in the established psychological construct need for cognition and is supported by multiple studies with over 1500 participants.

|  |  |  | Completely disagree | Largely disagree | Slightly disagree | Slightly agree | Largely Agree | Completely agree |
| --- | --- | --- | --- | --- | --- | --- | --- | --- |
| 1. | I like to occupy myself in greater detail with technical systems. |  |  |  |  |  |  |  |
| 2. | I like testing the functions of new technical systems. |  |  |  |  |  |  |  |
| 3. | I predominantly deal with technical systems because I have to. |  |  |  |  |  |  |  |
| 4. | When I have a new technical system in front of me, I try it out intensively. |  |  |  |  |  |  |  |
| 5. | I enjoy spending time becoming acquainted with a new technical system. |  |  |  |  |  |  |  |
| 6. | It is enough for me that a technical system works; I don’t care how or why. |  |  |  |  |  |  |  |
| 7. | I try to understand exactly how a technical system works. |  |  |  |  |  |  |  |
| 8. | It is enough for me to know the basic functions of a technical system. |  |  |  |  |  |  |  |
| 9. | I try to make full use of the capabilities of a technical system. |  |  |  |  |  |  |  |

Analysis

1. When entering the participants’ responses into a data file for the analysis, the responses should be coded as follows: completely disagree = 1, largely disagree = 2, slightly disagree = 3, slightly agree = 4, largely agree = 5, completely agree = 6.
2. Responses to the three negatively worded items (items 3, 6, 8) must be reversed (6 = 1, 5 = 2, 4 = 3, 3 = 4, 2 = 5, 1 = 6).
3. Finally, a mean score should be computed over all nine items.
