# Supplementary material for: A Technological Tool Aimed at Self-Care in Patients With Multimorbidity: Cross-Sectional Usability Study
Source: JMIR Hum Factors. 2024 Apr 5;11:e46811. doi: 10.2196/46811 (PMC11031692; doi:10.2196/46811)
Supplement: Multimedia Appendix 6 [file humanfactors_v11i1e46811_app6.docx]

Subgroup analysis considering patients with cognitive problems.

| TASKS | TOTAL  (n = 30) | No cognition problems  (n = 16) | Cognition problems  (n =14) |
| --- | --- | --- | --- |
| TASK 1 |  |  |  |
| Usability effectiveness |  |  |  |
| Number of patients completing the task, n (%) | 30 (100.0%) | 16 (100.0%) | 14 (100.0%) |
| Number of errors made, median (IQR) | 0.0 (0.0, 0.0) | 0.0 (0.0, 0.0) | 0.0 (0.0, 0.0) |
| Completes the task with personalized help, n (%) | 7 (23.3%) | 1 (6.2%) ^b^ | 6 (42.9%) ^b^ |
| Usability efficiency |  |  |  |
| Time to perform the task (seconds), median (IQR) | 18.5 (11.0, 30.0) | 13.5 (9.0, 18.5) ^a^ | 29.0 (20.0, 40.0) ^a^ |
| TASK 2 |  |  |  |
| Usability effectiveness |  |  |  |
| Number of patients completing the task, n (%) | 30 (100.0%) | 16 (100.0%) | 14 (100.0%) |
| Number of errors made, median (IQR) | 0.0 (0.0, 0.0) | 0.0 (0.0, 0.0) | 0.0 (0.0, 0.0) |
| Completes the task with personalized help, n (%) | 3 (10.0%) | 0 (0.0%) ^c^ | 3 (21.4%) ^c^ |
| Usability efficiency |  |  |  |
| Time to perform the task (seconds), median (IQR) | 15.5 (10.0, 26.0) | 11.0 (10.0, 19.5) | 24.0 (10.0, 40.0) |
| TASK 3 |  |  |  |
| Usability effectiveness |  |  |  |
| Number of patients completing the task, n (%) | 28 (93.3%) | 15 (93.8%) | 13 (92.9%) |
| Number of errors made, median (IQR) | 0.0 (0.0, 1.0) | 1.0 (0.0, 1.0) | 0.0 (0.0, 1.0) |
| Completes the task with personalized help, n (%) | 10 (33.3%) | 3 (18.8%) | 7 (50.0%) |
| Usability efficiency |  |  |  |
| Time to perform the task (seconds), median (IQR) | 57.0 (33.0, 90.0) | 53.5 (34.0, 80.0) | 66.0 (30.0, 90.0) |
| TASK 4 |  |  |  |
| Usability effectiveness |  |  |  |
| Number of patients completing the task, n (%) | 22 (73.3%) | 13 (81.2%) ^b^ | 9 (64.3%) ^b^ |
| Number of errors made, median (IQR) | 2.0 (0.0, 4.0) | 2.0 (0.0, 3.5) | 2.0 (0.0, 4.0) |
| Completes the task with personalized help, n (%) | 8 (26.7%) | 3 (18.8%) | 5 (35.7%) |
| Usability efficiency |  |  |  |
| Time to perform the task (seconds), median (IQR) | 182.5 (0.0, 280.0) | 176.0 (128.5, 255.0) | 186.0 (0.0, 320.0) |
| TASK 5 |  |  |  |
| Usability effectiveness |  |  |  |
| Number of patients completing the task, n (%) | 26 (86.7%) | 16 (100.0%) | 10 (71.4%) |
| Number of errors made, median (IQR) | 1.0 (0.0, 2.0) | 0.5 (0.0, 1.0) | 1.5 (0.0, 2.0) |
| Completes the task with personalized help, n (%) | 5 (16.7%) | 2 (12.5%) | 3 (21.4%) |
| Usability efficiency |  |  |  |
| Time to perform the task (seconds), median (IQR) | 20.0 (10.0, 50.0) | 20.0 (12.0, 27.5) | 33.0 (0.0, 75.0) |

^a^*P*=.001.

^b^*P*=.03.

^c^*P*=.09.
